# Rare SNPs in receptor tyrosine kinases are negative outcome predictors in multiple myeloma

**DOI:** 10.18632/oncotarget.9607

**Published:** 2016-05-26

**Authors:** Sarah Keppler, Susann Weiβbach, Christian Langer, Stefan Knop, Jordan Pischimarov, Miriam Kull, Thorsten Stühmer, Torsten Steinbrunn, Ralf Bargou, Hermann Einsele, Andreas Rosenwald, Ellen Leich

**Affiliations:** ^1^ Institute of Pathology, University of Würzburg, Würzburg, Germany; ^2^ Comprehensive Cancer Center Mainfranken (CCC MF), University Hospital Würzburg, Würzburg, Germany; ^3^ Department of Internal Medicine III, University Hospital Ulm, Ulm, Germany; ^4^ Department of Internal Medicine II, University Hospital Würzburg, Würzburg, Germany

**Keywords:** multiple myeloma, receptor tyrosine kinases, amplicon sequencing, rare SNP

## Abstract

Multiple myeloma (MM) is a plasma cell disorder that is characterized by a great genetic heterogeneity. Recent next generation sequencing studies revealed an accumulation of tumor-associated mutations in receptor tyrosine kinases (RTKs) which may also contribute to the activation of survival pathways in MM. To investigate the clinical role of RTK-mutations in MM, we deep-sequenced the coding DNA-sequence of *EGFR*, *EPHA2*, *ERBB3*, *IGF1R*, *NTRK1* and *NTRK2* which were previously found to be mutated in MM, in 75 uniformly treated MM patients of the “Deutsche Studiengruppe Multiples Myelom”. Subsequently, we correlated the detected mutations with common cytogenetic alterations and clinical parameters. We identified 11 novel non-synonymous SNVs or rare patient-specific SNPs, not listed in the SNP databases 1000 genomes and dbSNP, in 10 primary MM cases. The mutations predominantly affected the tyrosine-kinase and ligand-binding domains and no correlation with cytogenetic parameters was found. Interestingly, however, patients with RTK-mutations, specifically those with rare patient-specific SNPs, showed a significantly lower overall, event-free and progression-free survival. This indicates that RTK SNVs and rare patient-specific RTK SNPs are of prognostic relevance and suggests that MM patients with RTK-mutations could potentially profit from treatment with RTK-inhibitors.

## INTRODUCTION

Multiple myeloma (MM) is a malignant disease of plasma cells characterized by the accumulation of monoclonal plasma cells in the bone marrow [1, 2]. The development proceeds via monoclonal gammopathy of undetermined significance and smoldering myeloma to symptomatic MM and plasma cell leukemia [3, 4]. On the molecular level, MM shows a great genetic heterogeneity with different clonal subgroups [2, 5]. Primary genetic events in the development of MM lead to the immortalization of differentiated plasma cells and include chromosomal translocations involving the *IGH*-locus on chromosome 14 such as t(4;14), t(11;14), t(14;16), t(8;14) and hyperdiploidy [3, 4].

Moreover, the RAS/MAPK pathway, the JAK/STAT pathway and the PI3K/AKT pathway have previously been reported to be deregulated in MM, leading to an increased proliferation and survival of MM cells [6–11]. In recent next generation sequencing studies single nucleotide variants (SNVs) have been observed in these pathways with *NRAS* and *KRAS* being among the most frequently mutated genes [5, 12–15]. Identical SNVs or single gene mutations, however, rarely occur in a significant amount of cases, but different SNVs do accumulate in specific signaling pathways. For example, we recently defined a signaling network composed of RTKs, adhesion molecules and their effectors and observed a tumor-associated SNV pattern that predicted inter- and intra-individual pathway redundancy [14]. RTKs are cell-surface receptors that have a conserved structure consisting of an extracellular region containing the ligand-binding domain, a transmembrane domain and an intracellular region containing the tyrosine-kinase (TK) domain and additional regulatory regions [16]. Most RTKs are monomeric polypeptide chains in the absence of ligand-binding, with the exception of IGF1R which exists as a disulfide-linked dimer in the absence of a ligand [17, 18]. RTKs dimerize upon ligand binding leading to autophosphorylation of the TK-domain and subsequent binding and activation of downstream effectors triggering signaling pathways including the PI3K/AKT and the RAS/MAPK pathway subsequently leading to cell differentiation and proliferation [19–22]. However, while overexpression and mutations in the RTK *FGFR3* have been shown in MM, [23–25] no information exists on how SNVs in other RTKs can effect MM development and progression. Given that RTKs play an important role in tumorigenesis and treatment of several cancer entities, [21, 26–29] we thus focused on the six RTK genes *EGFR*, *EPHA2*, *ERBB3*, *IGF1R*, *NTRK1* and *NTRK2* that were previously described to be mutated in MM and deep-sequenced their coding DNA sequence (CDS) in biopsies of 75 primary MM cases of the “Deutsche Studiengruppe Multiples Myelom” (DSMM) taken at diagnosis.

While we focused on tumor-associated non-synonymous SNVs in our previous whole exome sequencing study, we here investigated tumor-associated SNVs and non-synonymous SNPs before and after exclusion of SNPs listed in 1000 genomes and/or dbSNP. Specifically, we correlated the occurrence of SNVs, common SNPs and rare patient-specific SNPs with common cytogenetic alterations and/or clinical parameters to further elucidate their role in the clinical course of MM.

## RESULTS

### Sequencing output, filtering and technical verification

The CDS of *EPHA2*, *ERBB3*, *IGF1R*, *NTRK1* and *NTRK2* were covered on average with 2407, 2668, 2942, 2216 and 2370 reads/sample, respectively, and the CDS of *EGFR*, except for the ligand binding and TK-domain, with 2204 reads/sample. The ligand-binding and TK-domain of *EGFR* were sequenced with the 454 GS Junior and had an average coverage of 159 reads/sample (Supplementary Table S1, Supplementary Figure S1). The *EGFR* ligand-binding and TK-domain of one patient (P41) were not covered and therefore Sanger sequenced. Exons of *EPHA2*, *NTRK1*, *IGF1R* and *EGFR* with low coverage (<10x) were additionally sequenced by Sanger sequencing. However, no mutations were detected in these exons.

**Figure 1:**
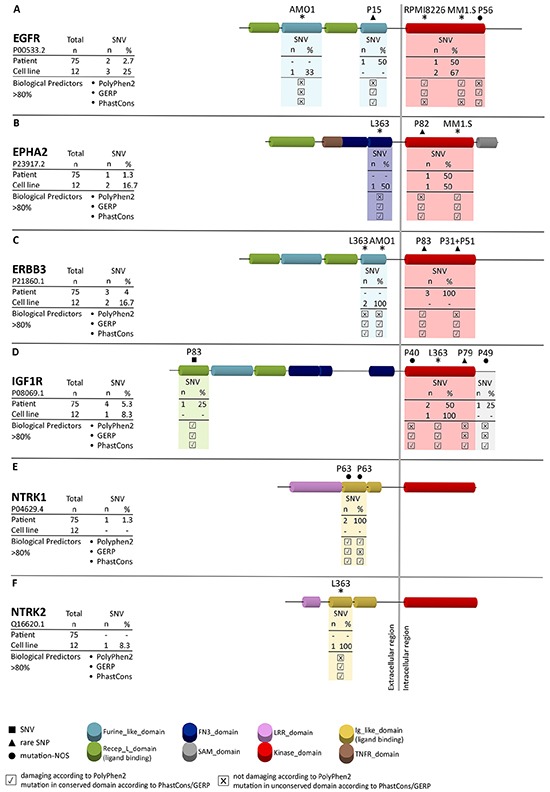
Frequency of mutations and affected regions in receptor tyrosine kinases Amplicon sequencing revealed novel mutations in the receptor tyrosine kinases *EGFR* **A.** *EPHA2* **B.** *ERBB3* **C.** *IGF1R* **D.** *NTRK1* **E.** *and NTRK2* **F.** SNVs, rare SNPs and mutations-not otherwise specified (mutations-NOS) are indicated by a square (■), triangle (▲) and circle (●), respectively, in combination with the corresponding patient number. Mutations in MM cell lines are indicated by asterisks. Functional predictions for each mutation in patients and MM cell lines are based on PhastCons and GERP (predicting the level of conservation) and PolyPhen2 (predicting structural changes). x indicates no influence of the mutation according to the functional predictor, ✔ indicates an influence of the mutation according to the functional predictor.

After first data processing, including read trimming, alignment and SNV calling, 156 mutations remained. 35 of the detected mutations were listed in the 1000 genome database and another 44 mutations in the dbSNPv134 and were excluded from the dataset. 26 mutations located in intronic regions, 2 mutations in untranslated regions, 1 mutation near a splice site and 18 synonymous mutations were removed. The remaining 30 mutations were verified by Sanger sequencing or high resolution melting assay (HRM) (Supplementary Figure S2). 9 mutations were only present in MM cell lines. All mutations that we previously detected in the 6 cell lines AMO1, INA6, JJN3, MM1.S, OPM2 and U266 by whole exome sequencing could also be detected in the current amplicon sequencing approach (Supplementary Table S2) [14]. 11 additional mutations that were detected by amplicon sequencing in primary MM cases could not be assessed by Sanger sequencing or high-resolution-melting (HRM). Of those, 9 mutations had a low variant allele frequency (VAF) and far lower quality parameters than the mutations that could be technically verified, strongly suggesting false positive SNVs (Supplementary Table S3). One mutation was identified using the 454 GS Junior platform and included in the dataset, resulting in a total of 11 distinct heterozygous mutations in 10 primary MM cases (Table 1). All 11 mutations were not listed in 1000 genomes or dbSNP, were non-synonymous, located in conserved domains and led to structural changes in 4 out of 11 cases according to Polyphen2. 5 out of the 11 mutations (45.5%) that occurred in 6 MM cases with corresponding normal tissue available were also detected in the corresponding normal sample. Contamination problems could be excluded since the same DNA samples served as templates in a previously published study which did not reveal mutations in the respective normal samples [30]. Moreover, the mutation frequency in the corresponding normal sample was comparable to that in the tumor sample and patient P83 was affected by a germline mutation in the TK-domain of *ERBB3* but a somatic mutation in the ligand-binding domain of *IGF1R* (Figure 1B, 1D). To separate those patient-specific SNPs from the common SNPs listed in the databases 1000 genomes and dbSNP, we defined them as rare SNPs which also correlates to the information received from over 60 000 individuals (allele frequency: 0 - 8.241×10^−5^) using the ExAC browser (http://exac.broadinstitute.org/). 1 out of the 11 mutations (1/11 [9%]) could be clearly defined as a tumor specific SNV and the remaining 5 mutations (5/11 [45%]) could not be clearly assigned to one of the two groups due to the lack of corresponding normal tissue. They were specified as mutations-not otherwise specified (NOS).

**Table 1:** Novel mutations detected in receptor tyrosine kinases

Gene	Chr.	Exon	Position (hg19)	Ref Base	Sample Alleles	Patient	VAF	cDNA Pos.	AA	PolyPhen2	PhastCons	GERP
EPHA2	1	6	16458908	A	A/G	P82	49.75	2080	TYR, HIS	benign	0.824	4.95
NTRK1	1	9	156841538	A	A/C	P63	53.29	751	ASN, HIS	probably-damaging	1	4.97
NTRK1	1	9	156841540	C	C/G	P63	54.21	753	ASN, LYS	probably-damaging	0.998	1.05
EGFR	7	13	55229263	G	G/C	P15	49.16	1570	VAL, LEU	benign	0.902	4.08
EGFR	7	27	55268938	A	A/G	P56	42.86(F) 35.42(R)	3004	MET, VAL	benign	1	5.65
ERBB3	12	21	56491703	G	G/T	P83	49.17	2595	GLN, HIS	probably-damaging	0.997	2.59
ERBB3	12	23	56492567	C	C/G	P31	24.38	2717	THR, SER	benign	0.999	5.29
ERBB3	12	23	56492567	C	C/G	P51	24.77	2717	THR, SER	benign	0.999	5.29
IGF1R	15	2	99251007	C	C/T	P83	9.24	311	THR, MET	probably-damaging	0.998	5.36
IGF1R	15	18	99482518	A	A/G	P40	26.43	3386	ASN, SER	benign	1	3.61
IGF1R	15	21	99500419	G	G/T	P79	65.62	3852	GLU, ASP	benign	1	0.576
IGF1R	15	21	99500663	A	A/C	P49	55.04	4096	THR, PRO	benign	1	2.46

Chr. = Chromosome, hg = human genome version 19, Ref Base = reference base, VAF = variant allele frequency, Pos.= position, AA = amino acid, TYR = tyrosine, HIS = histidine, ASN = asparagine, LYS = lysine, VAL = valine, LEU = leucine, MET = methionine, GLN = glutamine, THR = threonine, SER = serine, GLU = glutamic acid, ASP = aspartic acid, PRO = proline

### Accumulation of novel SNVs and rare SNPs in the kinase and ligand-binding domains of RTKs

All 11 verified SNVs and rare SNPs detected by amplicon sequencing in the CDS of the RTKs *EGFR*, *EPHA2*, *ERBB3*, *IGF1R*, *NTRK1* and *NTRK2* were not described previously according to the cosmic database (http://cancer.sanger.ac.uk/cosmic) and they collectively affected 10 out of 75 primary MM cases (13%) of the current study. Mutations that were previously detected in the cell lines L363, AMO1, MM1.S and U266 could be confirmed by the current amplicon approach (Figure 1A-1F) [14]. 4 rare SNPs and 2 mutations-NOS affected the TK-domains, 1 SNV and 2 mutations-NOS affected the ligand-binding domain, 1 rare SNP the furin-like domain and one mutation-NOS was found downstream of the TK-domain (Figure 1A-1F). The most affected RTK was *IGF1R* which was mutated in 4 out of 75 patients (5.3%), followed by *ERBB3* (4%), *EGFR* (2.7%), *EPHA2* and *NTRK1* (each 1.3%) as well as *NTRK2* (0%). More specifically, patients P82, P83/P51/P31 and P79 harbored a rare SNP in the TK-domain of *EPHA2*, *ERBB3* and *IGF1R*, respectively (Figure 1B-1D). Interestingly in the case of *ERBB3* P31 and P51 were affected by the same mutation, which in MM is a rarely-observed coincidence. Patients P56 and P40 were affected by a mutation-NOS in the TK-domain of *EGFR* and *IGF1R*, respectively (Figure 1A, 1D). Patient P83 had in addition a SNV in the ligand-binding domain of *IGF1R* and patient P63 harbored 2 mutations-NOS in the immunoglobulin-like domain of *NTRK1* (Figure 1D, 1E), which is responsible for ligand binding in members of the NTRK family [31]. The furin-like domain of *EGFR* was affected by a rare SNP in patient P15 and a mutation-NOS was found downstream of the TK-domain of *IGF1R* (P49) (Figure 1A, 1D). A mutation in the CDS of *NTRK2* was present in the MM cell line L363. However, no primary case was affected by SNVs, rare SNPs or mutations-NOS in *NTRK2* (Figure 1F). Among the cell lines that were not included in the whole exome sequencing approach but investigated in the current amplicon sequencing approach, only RPMI8226 was affected by a mutation in 1 of the 6 RTKs, namely in the TK-domain of *EGFR* (Figure 1A). All detected SNVs and rare SNPs in primary MM cases and all mutations detected in MM cell lines are located in conserved regions as indicated by the GERP and PhastCons scores and 4 out of the 11 mutations have an influence on the protein structure according to the bioinformatics predictor PolyPhen2 (Table 1; Figure 1).

In summary, we detected 11 novel non-synonymous heterozygous mutations in 10 primary MM cases that were predominantly located in the kinase or ligand-binding domain of the six RTK genes analyzed.

### Rare patient-specific RTK SNPs are not restricted to hematopoietic tissue

To answer the question whether the rare RTK SNPs are already present in the early phase of embryogenesis or may appear later during hematopoiesis, we investigated non-hematopoietic paraffin embedded normal tissue of three patients (P51 [*ERBB3*], P83 [*ERBB3*], P79 [*IGF1R*]). We could confirm the *ERBB3* SNP in the non-hematological normal samples of patients P51 (fatty skin of lower abdomen) and P83 (colon mucosa, liver, skin), as well as the *IGF1R* SNP in the non-hematological sample of patient P79 (colon) (Supplementary Figure S3). The occurrence of these SNPs in all non-hematological normal samples suggests that these mutations were inherited or acquired early in embryogenesis and did not arise later during hematopoiesis.

### RTK-mutations are not associated with *DIS3* mutations and common cytogenetic events

To further characterize the novel SNVs and rare SNPs detected in primary MM cases, we correlated all RTK-mutations with cytogenetic hallmarks that are commonly found in MM. Chromosomal translocations and genetic gains and losses were examined using FISH and the incidence of these cytogenetic events in our dataset was published previously (Supplementary Table S4) [30]. Mutations in the RTKs *EGFR*, *EPHA2*, *ERBB3*, *IGF1R* and *NTRK1* were not significantly associated with chromosomal losses of 13q14 and 17p13, chromosomal gains of 1q21, 9q34 and chromosomal translocations t(4;14), t(11;14), t(14;16), t(8;14) and t(14;20) (Table 2). Moreover, a correlation approach with mutated *DIS3*, one of the most frequently mutated genes in MM [12, 13] revealed no significant association between mutations in *DIS3* and mutations in the above mentioned RTKs (Table 2).

**Table 2:** Correlation of RTK mutations with cytogenetic events common in MM

Cytogenetic Parameters	WT, n=65	RTK_mut, n=10	*p*
13q deletion; no, yes	28;37	7;3	0.174
17p deletion; no, yes	53;12	8;2	1
1q gain; no, yes	42;23	6;4	1
9q gain; no, yes	38;27	3;7	0.17
t(4;14); no, yes	45;20	9;1	0.266
t(11;14); no, yes	50;15	7;3	0.695
t(14;16); no, yes	62;3	10;0	1
t(8;14); no, yes	62;3	9;0	1
t(14;20); no, yes	65;0	9;0	-
DIS3 mut; no, yes	56;9	10;0	0.598

WT = wild-type, RTK = receptor-tyrosine kinase, mut = mutated, n = number, p = p-value

### RTK-mutations are adversely correlated with survival

**Figure 2:**
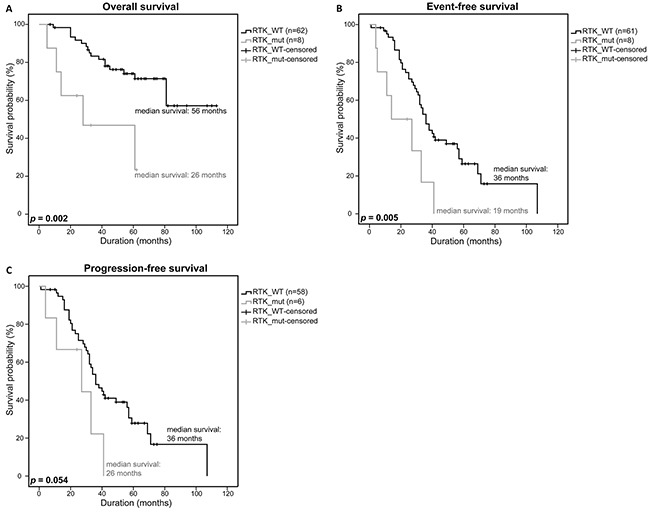
Clinical impact of RTK-mutations Overall survival **A.** event-free survival **B.** and progression-free survival **C.** of patients with a RTK SNV, rare SNP or SNV/rare SNP (RTK_mut) were compared to patients with a WT-RTK profile (RTK_WT) using a univariate analysis with log-rank test for significance (OS: RTK_WT n=62, RTK_mut n=8; EFS: RTK_WT n=61, RTK_mut n=8; PFS: RTK_WT n=58, RTK_mut n=6). *P*-values <0.05 were considered statistically significant.

Although the 11 RTK-mutations did not correlate with bad prognostic factors such as deletions of 17p13, 13q14 or t(4;14) and were not associated with a worse response to therapy (Supplementary Table S5 [32]), we observed a significantly lower survival rate in RTK-mutant patients. Patients without a mutation (n=62) had a median overall survival (OS) of 56 months, event-free survival (EFS) of 36 months and a progression-free survival (PFS) of 36 months while patients with a mutation (n=8) had a median OS of 26 months, an EFS of 19 months and a PFS of 26 months, respectively (*p*=0.002, *p*=0.005, *p*=0.054) (Figure 2A-2C).

**Figure 3:**
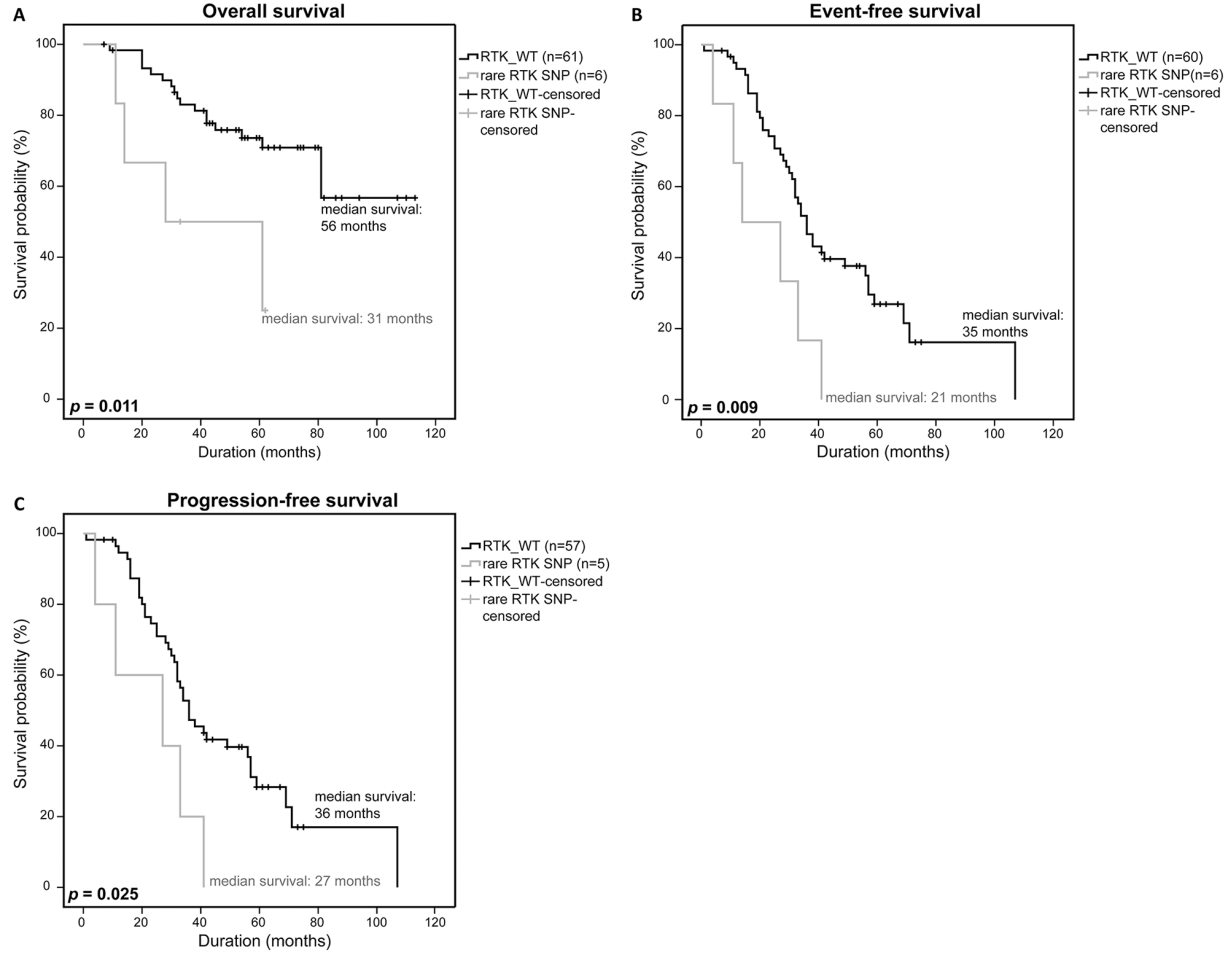
Clinical impact of rare RTK SNPs Overall survival **A.** event-free survival **B.** and progression-free survival **C.** of patients with a rare RTK SNP were compared to patients without a RTK-mutation (RTK_WT) using a univariate analysis with log-rank test for significance (OS: RTK_WT n=61, rare RTK SNP n=6; EFS: RTK_WT n=60, rare RTK SNP n=6; PFS: RTK_WT n=57, rare RTK SNP n=7). *P*-values <0.05 were considered statistically significant.

To test whether the rare RTK SNPs alone have a significant impact on survival as well, we furthermore restricted our analysis to the MM patients that were only affected by rare germline mutations. Survival statistics revealed a significantly lower OS (56 months vs. 31 months, *p*=0.011) and EFS (35 months vs. 21 months, *p*=0.009), and even a significantly lower PFS (36 months vs. 27 months, *p*=0.025) compared to the survival statistics that included all patients (Figure 3A-3C).

**Figure 4:**
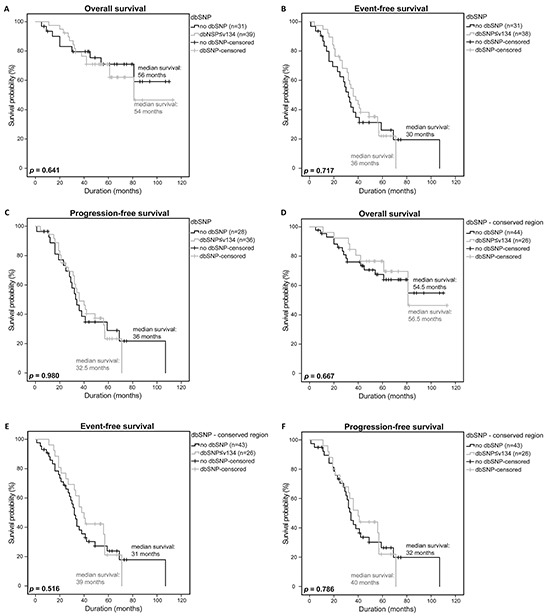
Clinical impact of RTK-mutations listed in dbSNP Overall survival **A.** event-free survival **B.** and progression-free survival **C.** of patients with a general SNP listed in dbSNP≤v134 were compared to patients without a SNP using a univariate analysis with log-rank test for significance (OS: no dbSNP n=31, dbSNP n=39; EFS: no dbSNP n=31, dbSNP n=38; PFS: no dbSNP n=28, dbSNP n=36). Overall survival **D.** event-free survival **E.** and progression-free survival **F.** of patients with a SNP in a conserved region and listed in dbSNP ≤v134 were compared to patients without a SNP in a conserved region (OS: no dbSNP n=44, dbSNP n=26; EFS: no dbSNP n=43, dbSNP n=26; PFS: no dbSNP n=43, dbSNP n=26). *P*-values <0.05 were considered statistically significant.

Due to the observation that the rare RTK SNPs that are not listed in dbSNPv134 and/or 1000 genomes have an influence on the survival of MM patients, we analyzed if common SNPs in general or common SNPs in conserved regions had an influence on survival as well. No significant differences in OS, EFS and PFS were observed in both analyses ((*p*=0.641, *p*=0.717, *p*=0.980) and (*p*=0.667, *p*=0.516, *p*=0.786); Figure 4A-4F). In addition, we stepwise analyzed our detected SNPs reported in different dbSNP builds (up to build 134) to see if SNPs reported in higher builds rather than those reported in lower builds could also be associated with the survival of MM patients. Moreover, we investigated SNPs that occurred in a maximum of 2 of the 75 samples and may thus also fulfill the criteria of a rare SNP, separately. However, neither these stepwise analysis approaches (n=12) nor the analysis of SNPs occurring in 2 samples or less showed a significant difference in survival of MM patients harboring SNPs of different builds, as compared to MM patients with no SNPs (OS: *p=*0.184-0.848, EFS: *p*=0.215-0.793, PFS: *p*=0.254-0.952; OS: *p*=0.532; EFS: *p*=0.953; PFS: *p*=0.739; respectively).

In summary, we could show that rare non-synonymous MM-associated SNPs that are located in conserved domains of the RTKs *EGFR*, *EPHA2*, *ERBB3*, *IGF1R* and *NTRK1* have a significant influence on OS, EFS and PFS in patients of the current study cohort and might thus be of prognostic relevance.

## DISCUSSION

Recent NGS studies revealed an accumulation of SNVs in RTKs, adhesion molecules and their downstream effectors and allowed to define a signaling network that was affected by at least one mutation in almost 100% of MM patients and more than one mutation in approximately 50% of MM patients [14].

This network also includes components of the MAPK and PI3K/AKT pathways, which can be activated by RTKs and have been reported to be consistently deregulated in MM [7, 19, 20, 33–35]. However, it is still a matter of debate as to whether recurrent mutations in key molecules of these pathways such as *RAS* and *BRAF* are of prognostic relevance. Depending on the study cohort and respective treatments, *KRAS* and *NRAS* mutations have adverse, beneficial or no effect on patient survival [6, 15, 36–38]. Mutations in *BRAF* were found to improve the response to broad acting drugs, though the clinical impact of the V600E mutation is not clear yet [39, 40]. In addition, oncogenic activation of the PI3K/AKT pathway can only partially be explained by previous investigations [11, 41, 42] and thus might also be explained by the occurrence of mutations in upstream molecules such as RTKs or other growth factors.

To further elucidate the role of RTK-mutations in MM, we deep-sequenced the CDS of the RTKs *EGFR*, *EPHA2*, *ERBB3*, *IGF1R*, *NTRK1* and *NTRK2* that were found to be mutated in previous studies and investigated the association of these mutations with common cytogenetic alterations and clinical parameters in a study cohort of 75 patients of the DSMM that were uniformly treated with bortezomib, autologous stem cell transplantation and high dose chemotherapy.

Interestingly, 9 out of 11 non-synonymous SNVs and rare patient-specific SNPs that were detected in RTKs in the current approach and that mainly affected *IGF1R* were located either in the TK- or ligand-binding domain and were not reported previously. Ligand binding leads to receptor dimerization, followed by auto-phosphorylation of the TK-domain and downstream signaling [19, 22]. Therefore, mutations in this domain could lead to discrepancies in ligand binding and subsequent downstream signaling. Additionally mutations in the TK-domain have been shown to have the potential to lead to constitutive phosphorylation and activation of RTKs [27, 43]. Both scenarios may trigger an adverse effect by a more sustained or accelerated downstream signaling promoting survival and proliferation of tumor cells and translating into a worse clinical outcome.

A significant association of RTK-mutations or rare RTK SNPs with common cytogenetic hallmarks of MM such as chromosomal gains of 1q21 or 9q34, chromosomal losses of 13q14 and 17p13, and the chromosomal translocations t(4;14), t(11;14), t(14;16), t(8;14) and t(14;20) was not observed (Table 2 and Supplementary Table S6).

Interestingly, however, our results - that need to be validated in a bigger patient cohort in a future study - strongly suggest that mutations in the RTKs have a significant impact on the survival of MM patients, specifically with regard to rare patient-specific SNPs (Figure 2, Figure 3). The occurrence of rare SNPs seem to be specific to the investigated RTKs based on the observation that such kind of rare SNPs were not detected in *DIS3* which was previously sequenced and analyzed in the same DSMM patient dataset [30]. The rare SNPs in RTKs that were identified in this study therefore seem to be non-random gene specific genetic changes that may serve as prognostic markers for MM patients.

Since we previously showed that del17p13 and del13q14 are adverse prognostic factors in the current dataset, [30] we performed a correlation approach that disregarded cases with these deletions to test if it is really the RTK-mutations that account for the significant differences in survival. Indeed, this analysis still revealed a significantly lower OS and EFS and a trend towards a lower PFS (del17p13: *p=*0.003, *p*=0.009, *p*=0.118, del13q14: *p=*0.003, *p*=0.043, *p*=0.129, respectively) (Supplementary Figure S4).

SNPs have been reported to influence cancer risk and progression in hematological and non-hematological malignancies, for example non-Hodgkin lymphoma, breast and prostate cancer [44–49]. However, only few genetic susceptibility factors have so far been described as risk factors for the development of MM, namely SNPs in *TERC*(3q26.2), *PSORS1C1*(6p21.33), *TNFRSF13B*(17p11.2), *CBX7*(22q13.1), *DNMT3A* (2p23.3), *ULK4*(3p22.1) and *CDCA7L*/*DNAH11*(7p15.3), as well as SNPs in *TNFα* and *LTα* [50–52].

If the rare RTK SNPs that we detected in the current analysis truly constitute predisposing risk factors in MM needs to be investigated in future studies. However, the presence of the rare RTK SNPs in non-hematopoietic tissue suggests that these mutations are either inherited or occur early in embryogenesis. This might indicate that rare RTK SNPs in MM are genetic events or predispositions that may act in concert with other mutations in MM rather than being initiators of tumorigenesis themselves.

To our knowledge, only the *TNFα* (−238) polymorphism has been described to be associated with improved survival in a study including MM patients treated with thalidomide [53] while there was only a trend towards an increased PFS in MM patients with the *TNFα/LTα* polymorphisms (−308, +252) treated with high dose chemotherapy [52]. The rare RTK SNPs that we identified in the current study are thus the first reported SNPs that were significantly associated with a worse outcome in MM patients treated with bortezomib, autologous stem cell transplantation and chemotherapy.

The oncogenic role of aberrant RTK expression and function as well as the successful use of RTK inhibitors, for example in RTK-mutant patients with glioblastoma or colorectal cancer have been previously described [27–29]. Therefore, it might be useful to screen patients with MGUS or MM at diagnosis for the presence of RTK-mutations, including rare patient-specific SNPs that may reveal potential susceptibilities to RTK-inhibitors.

## MATERIALS AND METHODS

### Human multiple myeloma cell lines and primary multiple myeloma samples

The study consisted of 75 primary MM samples from newly diagnosed symptomatic patients of the DSMM XI study and 12 MM cell lines. Primary samples were taken at initial diagnosis before treatment. Patients were treated with three cycles of combination therapy of bortezomib, dexamethasone and cyclophosphamide, subsequent stem cell mobilization, high-dose chemotherapy (HDC) and autologous stem cell therapy [54]. Clinical data was available for most patients (OS: n=70, EFS: n=69, PFS: n=64, response after bortezomib and HDC: n=67 and n=66). MM cells were isolated using CD138^+^ microbeads (Miltenyi Biotec, Bergisch Gladbach, Germany) as described previously [55]. Corresponding normal samples were collected from either bone marrow aspirates, peripheral blood or paraffin embedded tissue. The study was approved by the Ethics Committee of the Medical Faculty, University of Würzburg (reference number: 18/09, approval renewed: 09.03.2009, reference number AZ 76/13, date of approval: 18.04.2013) and University of Ulm (application number: 307/08, date of approval: 21.01.2009). The clinical trial is registered under ClinicalTrials.gov number NCT00833560.

The human MM cell lines AMO1, JJN3, KMS11, KMS12BM, L363, MOLP8, NCIH929, OPM2, RPMI8226 and U266 were obtained from the German Collection of Microorganisms and Cell Cultures (DSMZ, Braunschweig, Germany). The cell line MM1.S was purchased from LGC Biolabs (Wesel, Germany). The INA6 cell line was a kind gift from Prof. Martin Gramatzki (Kiel, Germany). The MM cell lines were cultured in RPMI1640 medium (Life Technologies, Darmstadt, Germany) containing 10% fetal bovine serum and 2mmol/l L-glutamine (PAN Biotech, Aidenbach, Germany). INA6 cells were supplemented with 2ng/ml human recombinant interleukin-6 and NCIH929 cells were supplemented with 0.05mM 2-mercapto-ethanol.

### Cytogenetic analysis

Chromosomal translocations t(4;14), t(11;14), t(14;16), t(8;14) and t(14;20), and genetic gains or losses of chromosome 1q21, 9q34, 13q14 and 17p13 were detected using fluorescence *in situ* hybridization (FISH) according to standard protocols [56, 57].

### Targeted resequencing of *EGFR, EPHA2, ERBB3, IGF1R, NTRK1* and *NTRK2*

A library of the coding DNA sequences of *EGFR*, *EPHA2*, *ERBB3*, *IGF1R*, *NTRK1* and *NTRK2* was prepared using 50ng DNA from each sample. The DNA was purified using the AllPrep DNA/RNA Mini Kit (Qiagen, Hilden, Germany). Target regions were amplified in a multiplex PCR using 224 tagged primer pairs, designed by Fluidigm (Supplementary Table S7), with the 48.48 Access Array^TM^ IFC and the FC1 Cycler System (Fluidigm, Amsterdam, The Netherlands) according to the manufacturer's protocol. Amplified target regions of each sample were pooled, diluted 1:100 and barcoded using the Access Array Barcode Library for Illumina Sequencers - 384 Single Direction (PN 100-4876, Fluidigm, Amsterdam, The Netherlands). Barcoded PCR products were analyzed on the 2100 Bioanalyzer (Agilent, Santa Clara, CA, USA), quantified using a Nanodrop spectrophotometer (Peqlab, Erlangen, Germany) and 20ng of each sample pool of one Access Array was pooled (Harvest sample pool). The Harvest sample pool was purified using AMPure-XP beads (BeckmanCoulter, Krefeld, Germany), analyzed on the 2100 Bioanalyzer and quantified using the Qubit system (Invitrogen, Darmstadt, Germany). An equal volume of each purified harvest sample pool of two performed Access Arrays was paired end sequenced on the MiSeq platform (Illumina, San Diego, USA) with a read length of 251bp.

The library preparation and subsequent pyrosequencing of the coding sequences of the *EGFR* ligand-binding and TK- domain were performed on the 454 GS Junior (Roche, Mannheim, Germany) with an average read length of 351bp as described previously (primer information: Supplementary Table S8) [30].

Sequence data is deposited at the European Genome-phenome Archive (EGA, http://www.ebi.ac.uk/ega/), hosted by the EBI (European Bioinformatics Institute), under accession number EGAS00001001665.

### Analysis of sequencing data

FASTQ files generated on the MiSeq platform were trimmed using the Cutadapt 1.3 software to remove adaptor and barcode sequences (Supplementary Figure S2). Trimmed reads were aligned to the reference genome (hg19) using the BWA tool. Processing of the alignment result as well as detection, identification and quantitation of mutations were performed using the standard setting of the GATK software package. SeattleSeq-annotation 137 web-client was used to annotate SNVs to the reference genome hg19 to characterize SNVs (http://snp.gs.washington.edu/SeattleSeqAnnotation137/). Mutations that were already listed in the 1000 genomes (http://www.1000genomes.org/) and the dbSNPv134 (http://www.ncbi.nlm.nih.gov/SNP/) databases were excluded from the dataset. In a manual filter step we selected for non-synonymous mutations in coding regions. As part of the SeattleSeq137 annotation the bioinformatic predictors PhastCons (−11.6 – +5.82) and GERP (0-1) for conservation and PolyPhen2 (benign, probably damaging, possibly damaging) for structural changes were used to classify the presumed functional relevance of detected SNVs. Assuming that the lowest score predicts no relevance (0%) and the highest score is equal to 100%, we chose a threshold of 80% for GERP and PhastCons.

The sequencing data output from the 454 GS Junior was analyzed as described previously [30]. Results of both approaches were combined and used for further investigations (Supplementary Figure S2).

VAF of mutations of the MiSeq data were calculated manually, VAFs of the GS Roche Junior were calculated by the AVA software. For technical validation, sequencing results of six cell lines (AMO1, INA6, JJN3, MM1.S, OPM2, U266) were compared with already existing whole-exome sequencing data (Supplementary Table S2) [14]. For technical verification, Sanger sequencing and HRM assays were performed. Unconfirmed mutations and mutations present only in cell lines were excluded from the statistical analysis. Mutations were manually assigned to protein domains using the NCBI protein database (http://www.ncbi.nlm.nih.gov/protein/).

### Sanger sequencing and high resolution melting assay

Newly detected and already described SNVs with VAF>20% were validated by Sanger sequencing in tumor and available corresponding normal sample according to standard protocols. Sequences were visualized using Chromas Lite (Technelysium, South Brisbane, Australia). SNVs with VAFs<20% were validated using an HRM assay as described previously [30].

Primers for Sanger sequencing and HRM were purchased from Integrated DNA Technologies (Leuven, Belgium) and Eurofins Genomics (Ebersberg, Germany) (Supplementary Table S9).

### Statistical analysis

Statistical analysis was performed using SPSS statistics software, version 23 (IBM, Ehningen, Germany). Kaplan-Meier curves were used for monovariate survival statistics in combination with log-rank tests for significance. Pearson chi-square and Fisher's exact-test were used for correlation of nominal variables. *P*-values < 0.05 were considered statistically significant.

## SUPPLEMENTARY FIGURES AND TABLES




















