# Supplementary material for: Rare SNPs in receptor tyrosine kinases are negative outcome predictors in multiple myeloma
Source: Oncotarget. 2016 May 26;7(25):38762–74. doi: 10.18632/oncotarget.9607 (PMC5122427; doi:10.18632/oncotarget.9607)
Supplement: Supplementary file 1 [file oncotarget-07-38762-s001.pdf]

Rare SNPs in receptor tyrosine kinases are negative outcome predictors in multiple myeloma

SUPPLEMENTARY FIGURES AND TABLES

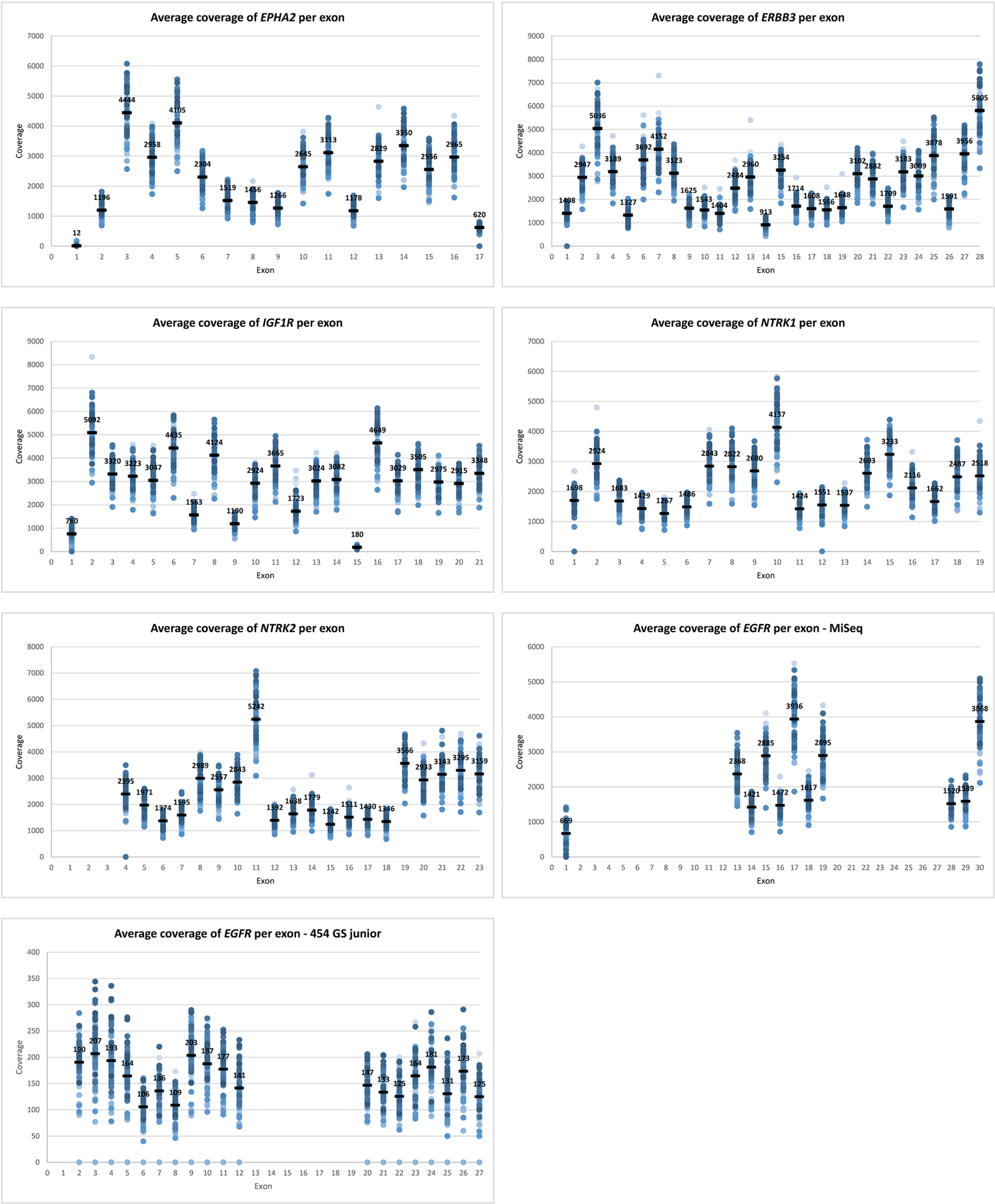

Supplementary Figure S1: Average coverage RTK exons. Coverage of the exons of the receptor-tyrosine kinases *EPHA2*, *ERBB3*, *IGF1R*, *NTRK1*, *NTRK2* and *EGFR*.

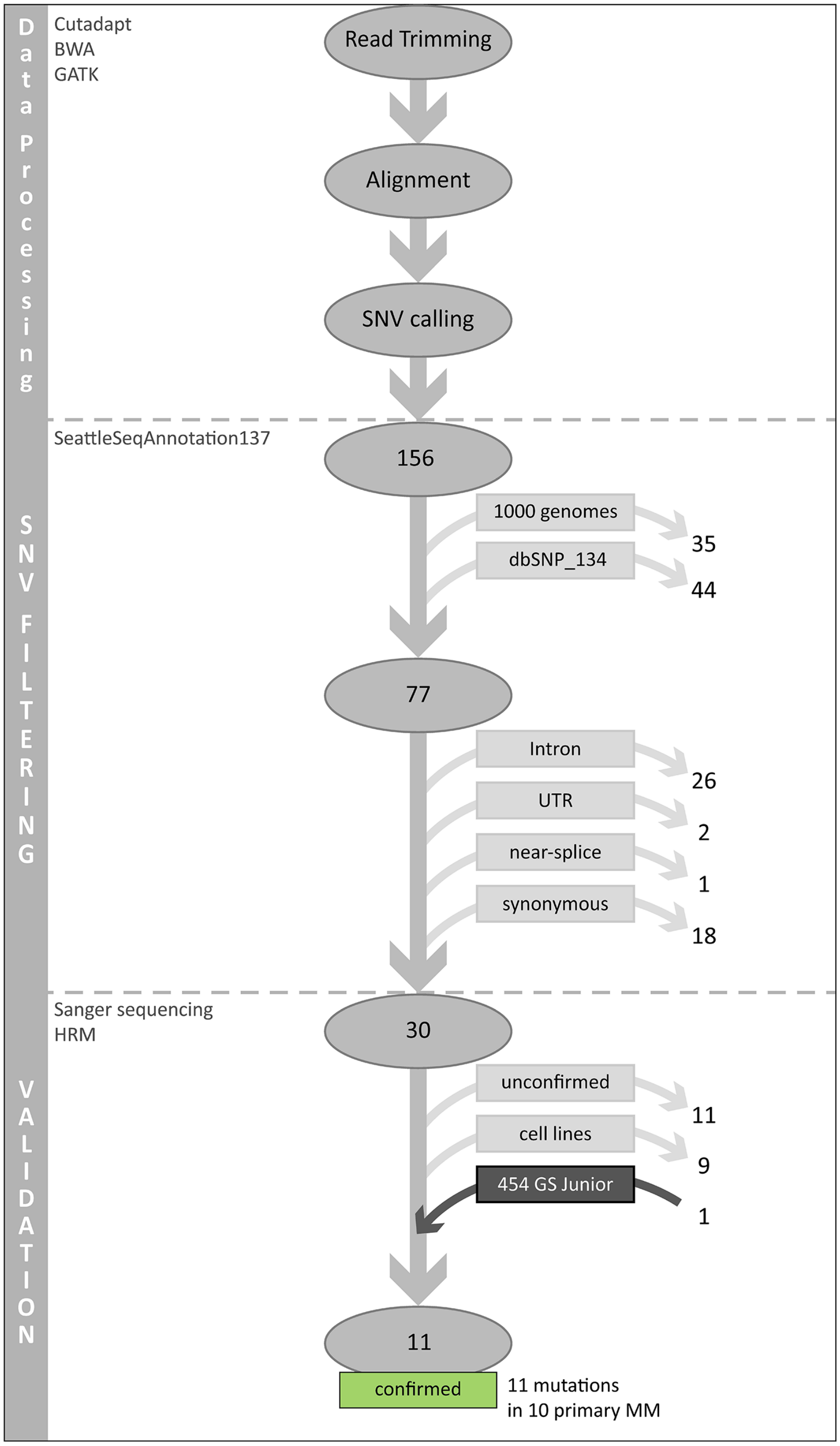

Supplementary Figure S2: Initial SNV-SNP filter step. Overview of the filtering strategy.

A

ERBB3 Chr.12 Ex.23 Pos.56492567C/G P51

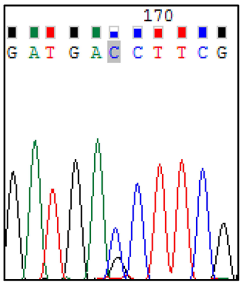

Fatty skin, lower abdomen

B

ERBB3 Chr.12 Ex.21 Pos.56491703 G/T P83

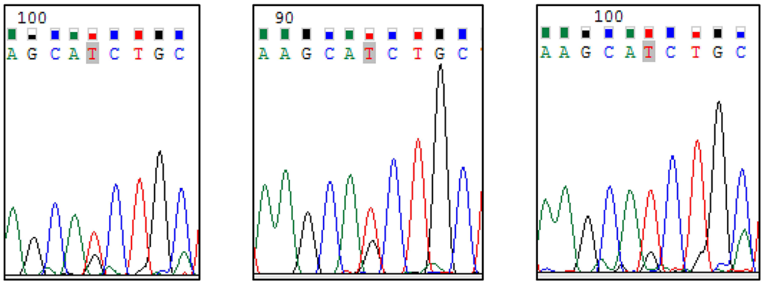

Colon mucosa Liver Skin

C

IGF1R Chr.15 Ex.21 Pos.99500419 G/T P79

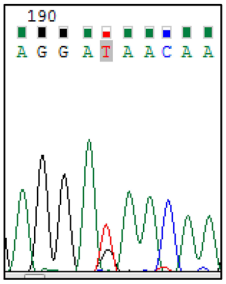

Colon

Supplementary Figure S3: Sanger sequencing of non-hematological normal samples. Non-hematological normal samples of P51 **A.** P83 **B.** and P79 **C.** from paraffin embedded tissue were sequenced using Sanger sequencing.

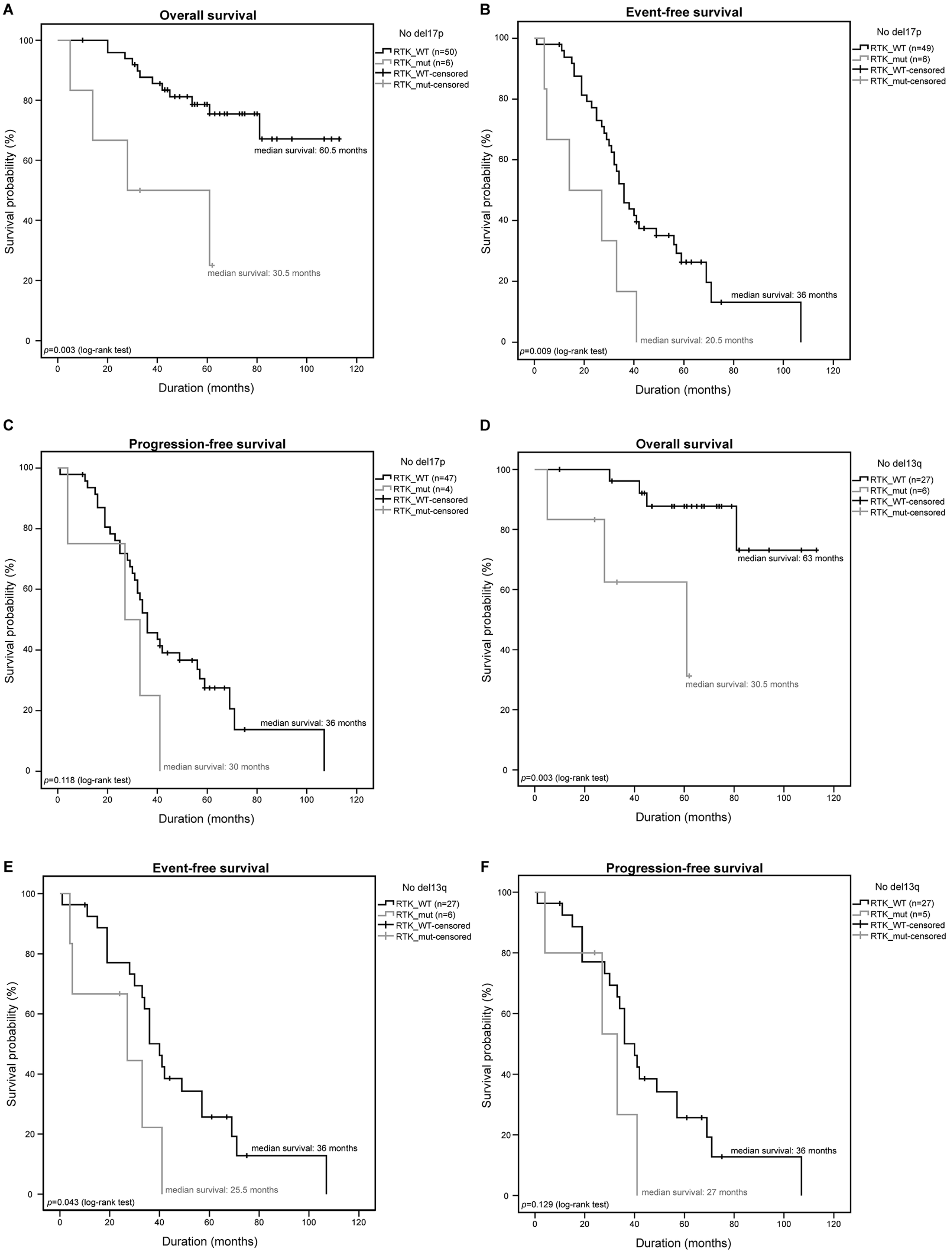

Supplementary Figure S4: Clinical impact of RTK-mutations without del17p or del13q. OS, EFS and PFs of patients with a RTK-mutation were compared to patients without a mutation (RTK_WT) after exclusion of patients with del17q **A-C.** and del13p **D-F.** using a univariate analysis approach with log-rank test for significance. *P*-values <0.05 were considered statistically significant.

Supplementary Table S1: Coverage RTK exons. Coverage of the exons of the receptor-tyrosine kinases *EPHA2*, *ERBB3*, *IGF1R*, *NTRK1*, *NTRK2* and *EGFR*.

See Supplementary File 1

Supplementary Table S2: Cell line mutations detected by amplicon and whole exom approach. Mutations detected using whole-exome sequencing and amplicon seqencing were compared.

See Supplementary File 2

Supplementary Table S3: RTK mutations with unclear validation status. Mutations detected by amplicon sequencing which could not be validated by HRM or sanger sequencing with different primer combinations and have therfore an unclear validation status.

See Supplementary File 3

Supplementary Table S4: Clinical data. Clinical information of patients from the current study.

See Supplementary File 4

Supplementary Table S5: Response to therapy. Response to therapy of patients with (Mut)and without (WT) RTK mutations were compared using pearson chi-square and fisher's exact-test. *P*-values < 0.05 were considered statistically significant.

See Supplementary File 5

Supplementary Table S6: Correlation of rare RTK SNPs with cytogenetics. Newly detected rare SNPs were correlated with cytogenetics common in MM.

See Supplementary File 6

Supplementary Table S7: Multiplex PCR primer. Sequences of tagged multiplex primers used to amplify target regions.

See Supplementary File 7

Supplementary Table S8: Primer 454 GS junior. Sequences of primers used.

See Supplementary File 8

Supplementary Table S9: Primer information sanger sequencing and HRM. Sequences of primers used for HRM and sanger sequencing to validate newly detected mutations.

See Supplementary File 9
